# MACC1 Down-Regulation Inhibits Proliferation and Tumourigenicity of Nasopharyngeal Carcinoma Cells through Akt/β-Catenin Signaling Pathway

**DOI:** 10.1371/journal.pone.0060821

**Published:** 2013-04-03

**Authors:** Fengjiao Meng, Hui Li, Huijuan Shi, Qingxu Yang, Fenfen Zhang, Yang Yang, Lili Kang, Tiantian Zhen, Sujuan Dai, Yu Dong, Anjia Han

**Affiliations:** 1 Department of Pathology, the First Affiliated Hospital and Zhongshan School of Medicine, Sun Yat-Sen University, Guangzhou, China; 2 Department of Pathology, Huizhou Municipal Central Hospital, Huizhou, China; Duke University Medical Center, United States of America

## Abstract

The present study was aimed at investigating the expression of metastasis-associated in colon cancer 1 (MACC1) in nasopharyngeal carcinoma (NPC), its relationship with β-catenin, Met expression and the clinicopathological features of NPC, and its roles in carcinogenesis of NPC. Our results showed that MACC1 expression was higher in NPC cells and tissues than that in normal nasopharyngeal cells and chronic inflammation of the nasopharynx tissues, respectively. MACC1 expression was closely related to the clinical stage (p = 0.005) and the N classification (p<0.05) of NPC. Significant correlations between MACC1 expression and Met expression (p = 0.003), MACC1 expression and β-catenin abnormal expression (p = 0.033) were found in NPC tissues. MACC1 knockdown dramatically inhibited cellular proliferation, migration, invasion, and colony formation, but induced apoptosis in NPC cells compared with the control group. Furthermore, MACC1 down-regulation inhibited phosphorylated-Akt (Ser473) and β-catenin expression in NPC cells, but phosphorylated-Erk1/2 expression was not altered. Further study showed that phosphotidylinsitol-3-kinase inhibitor downregulated β-catenin and Met expression in NPC cells. There was a significant relationship between MACC1 expression and phosphorylated-Akt expression (p = 0.03), β-catenin abnormal expression and phosphorylated-Akt expression (p = 0.012) in NPC tissue, respectively. In addition, Epstein Barr virus-encoded oncogene latent membrane protein 1 upregulated MACC1 expression in NPC cells. Our results firstly suggest that MACC1 plays an important role in carcinogenesis of NPC through Akt/β-catenin signaling pathway. Targeting MACC1 may be a novel therapeutic strategy for NPC.

## Introduction

Nasopharyngeal carcinoma (NPC) is relatively rare in the Western world, but more common in Southern China and Southeast Asia, with an annual incidence rate of about 20 per 100,000 people in endemic areas[1]. The epidemiologic evidence implies that environment risk factors, Epstein- Barr virus (EBV) infection and genetic susceptibility play crucial roles in carcinogenesis of NPC[2]. EBV-encoded oncogene latent membrane protein 1 (LMP1) is an important oncogene, which is involved in the activation of signal transduction pathways, such as the nuclear factor-κB, signal transducer and activator of transcription, and activator protein 1, which mediates various biological functions including proliferation, apoptosis, differentiation, and metastasis[3].

The Wnt/β-catenin signaling pathway is tightly regulated and has important functions in development, tissue homeostasis, and regeneration. Oncogenic activation of the Wnt/β-catenin signaling pathway by mutation in adenomatous polyposis coli or β-catenin, which results in the cytoplasmic and nuclear β-catenin accumulation and in β-catenin/T-cell factor (TCF) 4—regulated transcription of TCF target genes such as cyclin D1 and c-Myc, is mandatory for the initial neoplastic transformation of intestinal epithelium[4]. Our previous study has reported that LMP1 increases nuclear β-catenin accumulation and its transcriptional activity in NPC[5]. β-catenin knockdown dramatically inhibited cellular growth, migration and invasion, but induced apoptosis of NPC cells[6].

Metastasis-associated in colon cancer-1(MACC1), a newly identified key regulator of hepatocyte growth factor(HGF)-MET signaling, predicts colon cancer metastasis[7], [8]. Recently, MACC1 expression has been found in lung cancer[9], [10], hepatocellular carcinoma[11], ovarian carcinoma[12], and gastric carcinoma[13]. Overexpression of MACC1 associates with the progression of these carcinomas and prognosis of the patients with these carcinomas. To our knowledge, there is no report on MACC1 expression and its roles in carcinogenesis of NPC in the literature.

Our current paper firstly reported MACC1 expression, its relationship with β-catenin, Met expression, and the clinicopathological features of NPC. Moreover, MACC1 knockdown dramatically inhibited cellular proliferation, migration, invasion, and colony formation, but induced apoptosis in NPC cells, which might through down-regulating phosphorylated-Akt (p-Akt) and β-catenin expression.

## Materials and Methods

### Cell lines and cell culture

All NPC cell lines kindly supplied by the Cancer Center of Sun Yat-sen University, China were maintained in RPMI 1640 medium containing 10% fetal bovine serum, 100 units/ml of penicillin, and 0.1 mg/ml of streptomycin (Sigma, St. Louis, MO). CNE1 is an EBV-negative and well differentiated human NPC cell line, CNE2, HNE-1, and SUNE1 are EBV-negative and poorly differentiated human NPC cell line. C666-1 is EBV-positive poorly differentiated human NPC cell line. NP69 is normal nasopharyngeal cell line kindly supplied by the Cancer Center of Sun Yat-sen University, China. Small interfering RNA (siRNA) duplexes were synthesized and purified by Ribobio Inc. (Guangzhou, Guangdong, China). The siRNA sequences for MACC1 used were: sense 5′- CAC CAU AGC UUG CAA AGU A dTdT-3′, antisense 5′UAC UUU GCA AGC UAU GGU G- dTdT-3′. The siRNA sequences for Met used were: sense 5′ CCA AUG ACC UGC UGA AAU U dTdT 3′, antisense 5′AAU UU C AGC AGG UCA UUG G- dTdT- 3′. Transfection of siRNAs was done using Lipofectamine 2000 reagent (Invitrogen Co., Carlsbad, CA) according to the manufacturer's instructions.

### Patient information and tissue specimens

A total of 85 samples of paraffin-embedded, archived NPC tissues and 24 samples of chronic inflammation of the nasopharynx between 1998 to 2003 were collected from Department of Pathology, the first Affiliated Hospital, Sun Yat-sen University, China. For the research purposes of these clinical materials, prior patient's consents and approval from the Institutional Research Ethics Committee were obtained. No patients had received chemotherapy or radiotherapy before biopsy. The histopathology of the disease was determined by two pathologists according to the criteria of the World Health Organization classification of Head and Neck tumors (2005)[14]. Seventy-eight cases were undifferentiated carcinoma of nasopharyngeal type, four cases were differentiated non-keratinizing carcinoma, and three cases were keratinizing squamous cell carcinoma. Sixty-four patients were male, twenty-one patients were female. The average age was 48.68 years (age ranged from 11 to 67 years). Clinical staging was done according to the Union for International Cancer Control (UICC) classification[15]. Detailed clinical information about these patients, including gender, age, histological type, clinical stage, T classification, N classification, distant metastasis status, therapy modality, and radiation dose is summarized in Table 1.

**Table 1:** Association of MACC1 expression with the clinicopathological features of nasopharyngeal carcinoma patients.

Characteristics		MACC1 expression		P Value
		Low (%)	High (%)	
Age(years)	>46	19 (22.4)	29 (34.1)	0.078
	≤46	8 (9.4)	29 (34.1)	
Gender	Male	20 (23.5)	44 (51.8)	0.859
	Female	7 (8.2)	14 (16.5)	
Histological type (WHO)	UCNT	23 (27.1)	55 (64.7)	0.288
	DNKC	2 (2.4)	2 (2.4)	
	KSCC	2 (2.4)	1 (1.2)	
TNM staging	I	5 (5.9)	0 (0.0)	**0.005**
	II	7 (8.2)	12 (14.1)	
	III	8 (9.4)	28 (32.9)	
	IV	7 (8.2)	18 (21.2)	
T classification	T_1_	8 (9.4)	7 (8.2)	0.217
	T_2_	5 (5.9)	18 (21.2)	
	T_3_	7 (8.2)	18 (21.2)	
	T_4_	7 (8.2)	15 (17.6)	
N classification	N_0_	9 (10.6)	16 (18.8)	**0.040**
	N_1_	16 (18.8)	21 (24.7)	
	N_2_	2 (2.4)	16 (18.8)	
	N_3_	0 (0.0)	5 (5.9)	
Distant metastasis	M_0_	27 (31.8)	56 (65.9)	1.000
	M_1_	0 (0.0)	2 (2.4)	
Therapy	Radiotherapy	21 (24.7)	44 (51.8)	0.846
Chemotherapy& radiotherapy		6 (7.1)	14(16.5)	
Radiation dose of primary site	≤68.78	13 (15.3)	16 (18.8)	0.063
	>68.78	14 (16.5)	42 (49.4)	
Radiation dose of cervical lymph node	59.70	10 (11.8)	18 (21.2)	0.584
	>59.70	17 (20.0)	40 (47.1)	
β-catenin expression	Normal	18 (21.2)	19 (22.4)	**0.003**
	Abnormal	9 (10.6)	39 (45.9)	
Met expression	Low	11 (12.9)	11 (12.9)	**0.033**
	High	16 (18.8)	47 (55.3)	
LMP1 expression	Negative	16(18.8)	23(27.1)	0.091
	Positive	11(12.9)	35(41.2)	

**Abbreviation:** DNKC: differentiated non-keratinizing carcinoma; KSCC: keratinizing squamous cell carcinoma; UCNT: undifferentiated carcinoma of nasopharyngeal type.

### Real-time PCR analysis

As described previously[16], total RNA from cells was extracted using the Trizol reagent (Invitrogen,Carlsbad,CA, USA). Real-Time PCR was done according to the manufacturer(Bio-Rad Laboratories, Hercules, CA)'s instructions. The primer sequences used for MACC1 were purchased from TIANGEN BIOTECH (Beijing, China) CO., LTD. and followed: forward: 5′ TTC TTT TGA TTC CTC CGG TGA; reverse: 5′ ACT CTG ATG GGC ATG TGC TG. The primer sequences used for β-catenin were followed: forward: 5′ TTG AAA ATC CAG CGT GGA CA; reverse: 5′ TCG AGT CAT TGC ATA CTG TC. The primer sequences used for Met were followed: forward: 5′ TGT TCG ATA TTC ATC ACG GC; reverse: 5′ GCA TTT TTA CGG ACC CAA TC. The primer sequences used for c-Myc were followed: forward: 5′ GGC CCC CAA GGT AGT TAT CCT T; reverse: 5′ CGT TTC CGC AAC AAG TCC TCT. The geometric mean of housekeeping gene GAPDH was used to normalize the variability at mRNA expression levels.

### Western blot analysis

As described previously[16], the cell pellet was washed twice with ice-cold phosphate buffered saline (PBS) and lysed with lysis buffer. 30μg of protein was loaded and separated in 12% sodium dodecyl sulfate polyacrylamide gel electrophoresis ( SDS-PAGE) gel and transferred to polyvinylidine difluoride membranes (Millipore, Bedford, MA). The following antibodies were used to probe the alterations of protein: MACC1(Abcam, Cambridge, UK), Met(Abcam, Cambridge, UK), β-catenin (Santa Cruz Biotechnology, Santa Cruz, CA), c-Myc (Santa Cruz Biotechnology, Santa Cruz, CA), caspase-3 (Santa Cruz Biotechnology, Santa Cruz, CA), Akt (pan) (Cell Signaling Technology, Inc, Danvers, MA), phosphorylated-Akt (Ser473) (Cell Signaling Technology, Inc, Danvers, MA), phosphorylated-Erk1/2 (Cell Signaling Technology, Inc, Danvers, MA) and LMP1 mouse monoclonal antibody (DAKO). Signal was detected by enhanced chemoluminescence techniques (Amersham Life Science, Piscataway, NJ). GAPDH (Sigma, St. Louis, MO) was used as loading control.

### Cell proliferation assay

As described previously[6], 7×10^3^ cells were seeded in each well of 96-well plate and incubated overnight. The medium was removed. 100 µl of full assay medium with the final concentration of 100 nM MACC1 siRNA was added to each well, scramble siRNA, mock(adding only Lipofectamine 2000 reagent), or untreated cells were used as control group. All groups were triplicated. After 24 hour, 48 hour, and 72 hour transfection, cell proliferation was determined by 3-(4,5-dimethyl thiazol-2-yl) -2,5- diphenyl tetrazolium bromide (MTT) assay (CellTiter 96 Non- Radioactive Cell Proliferation Assay Kit, Promega Corporation, Madison, WI).

### Analysis of apoptosis

6×10^5^ cells were seeded in 6-well plates and incubated overnight till 50%–60% confluence. MACC1 siRNA was added to medium at a final concentration of 100 nM. Scramble siRNA, mock, or untreated cells were used as control group. The cells were transfected and harvested at 48 hour, washed in cold PBS, then fixed with 80% ethanol for 8 hours at 4°C , then stained with propidium iodide buffer (50 mg/ml propidium iodide, 0.1% sodium citrate, and 0.1%Triton X-100) for 3 hours at 4°C. 2×10^4^ cells were analyzed for apoptosis using a Becton Dickinson FACScan (Becton Dickinson Immunocytometry Systems, San Jose, CA). The percentage of apoptotic cells was quantified using Cell Quest software. This experiment was triplicated independently.

### Scratch wound assay

Cells transfected with MACC1 siRNA were plated in a 24-well plate in triplicates. Scramble siRNA, mock or untreated cells were considered as negative control group. When cells were grown to confluency, vertical scratches were then made using a 200 µl plastic filter tip to create a ‘wound’ of approximately 200 µm in diameter. To eliminate dislodged cells, culture medium was removed and wells were washed with PBS. The number of new cells that had moved into the scratch was counted at 24 hour and 48 hour and digital images were taken until the cells filled the scratch wound on an inverted microscope.

### Transwell matrix penetration assay

3×10^4^ cells transfected with MACC1 siRNA or scramble siRNA for 48 hours were plated into the top side of polycarbonate Transwell filter coated with matrigel in the upper chamber of the BioCoatTM Invasion Chambers (BD, Bedford, MA) and incubated at 37°C for 22 hours, followed by removal of cells inside the upper chamber with cotton swabs. Migratory and invasive cells on the lower membrane surface were fixed in 1% paraformaldehyde, stained with hematoxylin, and counted (Ten random 100×fields per well). Cell counts were the mean number of cells per field of view. Three independent experiments were performed and the data were presented as mean ± standard deviation (SD).

### Colony formation assay

2×10^2^ cells were plated onto 60 mm plates after 48 hour transfection and cultured for 12 days. The colonies were stained with 1% crystal violet for 30 seconds after fixation with 10% formaldehyde for 5 minutes. Plates were scored for the number of visible colonies. All experiments were performed in triplicates.

### Immunohistochemistry staining

The sections were deparaffinized, rehydrated in serially graded ethanol, and heated in citric buffer (pH 6.0) once for 5 minutes in a microwave oven for antigen retrieval. They were then washed with distilled water, blocked with 3% hydrogen peroxide and incubated with the primary antibodies including MACC1(Abcam, Cambridge, UK), Met (Abcam, Cambridge, UK), β-catenin (Santa Cruz Biotechnology, Santa Cruz, CA) , LMP1 mouse monoclonal antibody (DAKO), and phosphorylated-Akt (Ser473) (Cell Signaling Technology, Inc, Danvers, MA) at 4°C for 12 hours. After washing with a 0.01 mol /L concentration of PBS, the sections were incubated with EnVision-HRP secondary antibody (Dako, Carpinteria, CA) for 30 minutes at room temperature, washed with a 0.01 mol /L concentration of PBS, stained with 0.5% diaminobenzidine and counterstained with Mayer's haematoxylin, then air dried, and mounted with glycerol gelatin.

### Evaluation of immunohistochemistry staining

Immunohistochemical staining was independently assessed by two researchers who were unaware of the specific proteins being assessed or their putative role in NPC. The degree of MACC1, Met and p-Akt immunostaining was based on both the proportion of positively stained tumor cells and intensity of staining. The proportion of tumor cells was scored as follows: 0 (no positive tumor cells), 1 (<10% positive tumor cells), 2 (10–50% positive tumor cells), and 3 (>50% positive tumor cells). Staining intensity was graded according to the following criteria: 0 (no staining); 1 (weak staining  =  light yellow), 2 (moderate staining  =  yellow brown), and 3 (strong staining  =  brown). Staining index was calculated as the staining intensity score×the proportion score. We evaluated the expression of MACC1, Met and p-Akt in NPC specimen by determining the staining index, which scores as 0, 1, 2, 3, 4, 6, and 9. The staining index score of 4 (the cutoff point) was used to distinguish between low and high expression of MACC1, Met and p-Akt. The staining of β-catenin was scored according to Maruyama's method[17]. When more than 70% of carcinoma cells were positively stained for membranous β-catenin, the cells was classified as β-catenin normal expression; If more than 10% of carcinoma cells were positively stained for cytoplasm or nuclei was regards as β-catenin abnormal expression.

### Statistical analyses

All statistical analyses were performed using SPSS 16.0 statistics software. Student t test was used to compare the levels of cellular proliferation, apoptosis, migration, invasion, and colony formation between different groups. Chi-square test and Fisher exact test were used to compare the levels of MACC1, Met, β-catenin, p-Akt and LMP1 expression with different groups and various clinicopathological parameters. The correlation of MACC1, Met and β-catenin expression was analyzed by Spearman's correlation coefficients. p<0.05 was set to be statistically significant.

## Results

### MACC1 expression in NPC cell lines and tissues

**Figure 1:**
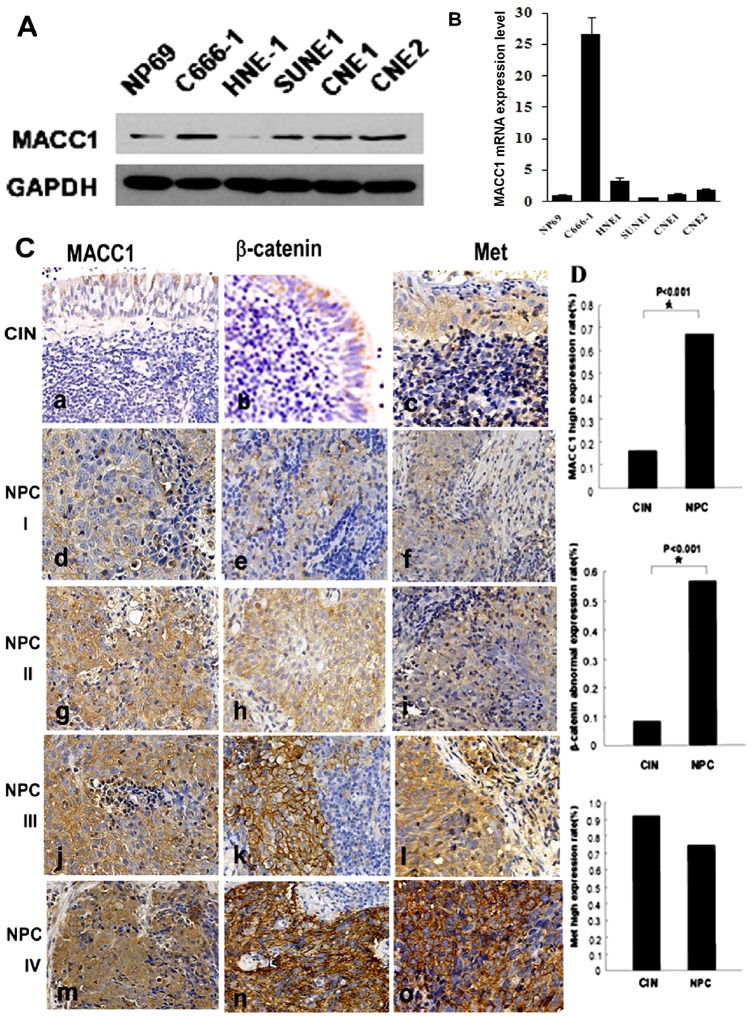
MACC1 protein expression in NPC cell lines (C666-1, HNE1, SUNE-1, CNE1 and CNE2) and normal nasopharyngeal cell (NP69) by western blot analysis(A); MACC1 mRNA expression in NPC cell lines and normal nasopharyngeal cell (NP69) by real-time PCR analysis (B); MACC1, β-catenin and Met expression in NPC and chronic inflammation of the nasopharynx (CIN) tissues by immunohistochemistry staining(C-D). AEG-1, β-catenin and Met expression was shown in CIN (a, b, c) and NPC with clinical stage I (d, e, f), II (g, h, i), III (j, k, l), and IV(m, n, o). Immunohistochemistry staining ×200(C); MACC1 and β-catenin expression was higher in NPC than that in chronic inflammation of the nasopharynx, respectively *p<0.001. There was no significant difference between Met expression in NPC and chronic inflammation of the nasopharynx (D).

MACC1 expression was higher in NPC cell lines including C666-1, SUNE-1, CNE1, and CNE2 except for HNE-1 than normal nasopharyngeal cells NP69 by western blot analysis. Moreover, we found that MACC1 expression was much higher in EBV-positive NPC cell line C666-1 than other EBV-negative NPC cell lines (Figure 1A). Meanwhile, MACC1 mRNA expression was much higher in NPC cell lines including C666-1, HNE-1, and CNE2 except for CNE1 and SUNE1 than normal nasopharyngeal cells NP69 by Real-time PCR analysis. Interestingly, MACC1 mRNA expression was dramatically higher in EBV-positive NPC cell line C666-1 than other EBV-negative NPC cell lines (Figure 1B).

In our series, MACC1 positive signals were mostly found in the cytoplasm of NPC cells, with only a minority of NPC cells stained in the nucleus by immunohistochemistry staining. Of 85 samples of paraffin-embedded NPC tissues, 58 samples (68%, 58/85) were MACC1 high expression, 27 samples (32%, 27/85) were MACC1 low expression. However, only 4 samples (16.7%, 4/24) were MACC1 high expression in chronic inflammation of the nasopharynx. MACC1 expression was significantly higher in NPC than that in chronic inflammation of the nasopharynx (p<0.01, Figure 1C-D).

### The relationship between MACC1 expression and β-catenin, Met LMP1, p-Akt expression and the clinicopathological features of NPC

Statistical analyses were done to examine the correlation between MACC1 expression and the clinicopathological characteristics of NPC. As shown in Table 1, the expression level of MACC1 protein was significantly related to UICC stage (p = 0.005) and the N classification (p<0.05). However, no significant association of MACC1 expression with gender, age, histological type, T classification, M classification, therapy modality and radiation dose was found.

As shown in Figure 1D, of 85 NPC cases, 48 cases (56.5%, 48/85) were β-catenin abnormal expression, 37 cases (43.5%, 37/85) were β-catenin normal expression by immunohistochemistry staining. However, there were only 2 cases (8.3%, 2/24) with β-catenin abnormal expression in chronic inflammation of the nasopharynx. Abnormal expression level of β-catenin was significantly higher in NPC than that in chronic inflammation of the nasopharynx (p<0.01). However, there was no significant association of β-catenin expression with gender, age, histological type, T classification, N classification, M classification, therapy modality and radiation dose (data not shown). Sixty three cases (74.1%, 63/85) and 22 cases (91.7%, 22/24) were Met high expression in NPC and chronic inflammation of the nasopharynx, respectively. In addition, Met expression was also found in stromal lymphocytes and plasma cells. There was no significant difference between Met expression in NPC and chronic inflammation of the nasopharynx (p>0.05, figure 1D).

As shown in Table 1, positive correlation between MACC1 expression and β-catenin abnormal expression was found in NPC (p = 0.003). Meanwhile, there was a significantly positive correlation between MACC1 expression and Met expression in NPC (p = 0.033). However, there was no significant association between β-catenin abnormal expression and Met expression (p>0.05).

LMP1 expression was located on the cell membrane and/or cytoplasm. In our series, LMP1 expression was found in 46 cases (54.1%) of NPC. p-Akt expression was located in the cytoplasm. p-Akt high expression was found in 43 cases (50.6%) of NPC. There was no significant association between LMP1 expression and MACC1 expression (p>0.05) although we noticed LMP1-positive group had higher MACC1 high expression than that in LMP1-negative group (Table 1). There was significant relationship between MACC1 expression and p-Akt expression (p = 0.03), β-catenin abnormal expression and p-Akt expression (p = 0.012) in NPC tissue, respectively. However, there was no significant relationship between Met expression and p-Akt expression (p = 0.121) (Table 2).

**Table 2:** The relationship between p-Akt expression and MACC1, β-catenin, and Met expression in nasopharyngeal carcinoma tissue.

Variables		p-Akt expression		P value
		Low (%)	High (%)	
MACC1 expression	Low	18 (21.2)	9 (10.6)	0.030
	High	24 (28.2)	34 (40.0)	
β-catenin expression	Normal	24 (28.2)	13 (15.3)	0.012
	Abnormal	18 (21.2)	30 (35.3)	
Met expression	Low	14 (16.5)	8 (9.4)	0.121
	High	28 (32.9)	35 (41.2)	

### MACC1 knockdown dramatically inhibited proliferation, migration, invasion and colony formation, but induced apoptosis in NPC cells

**Figure 2:**
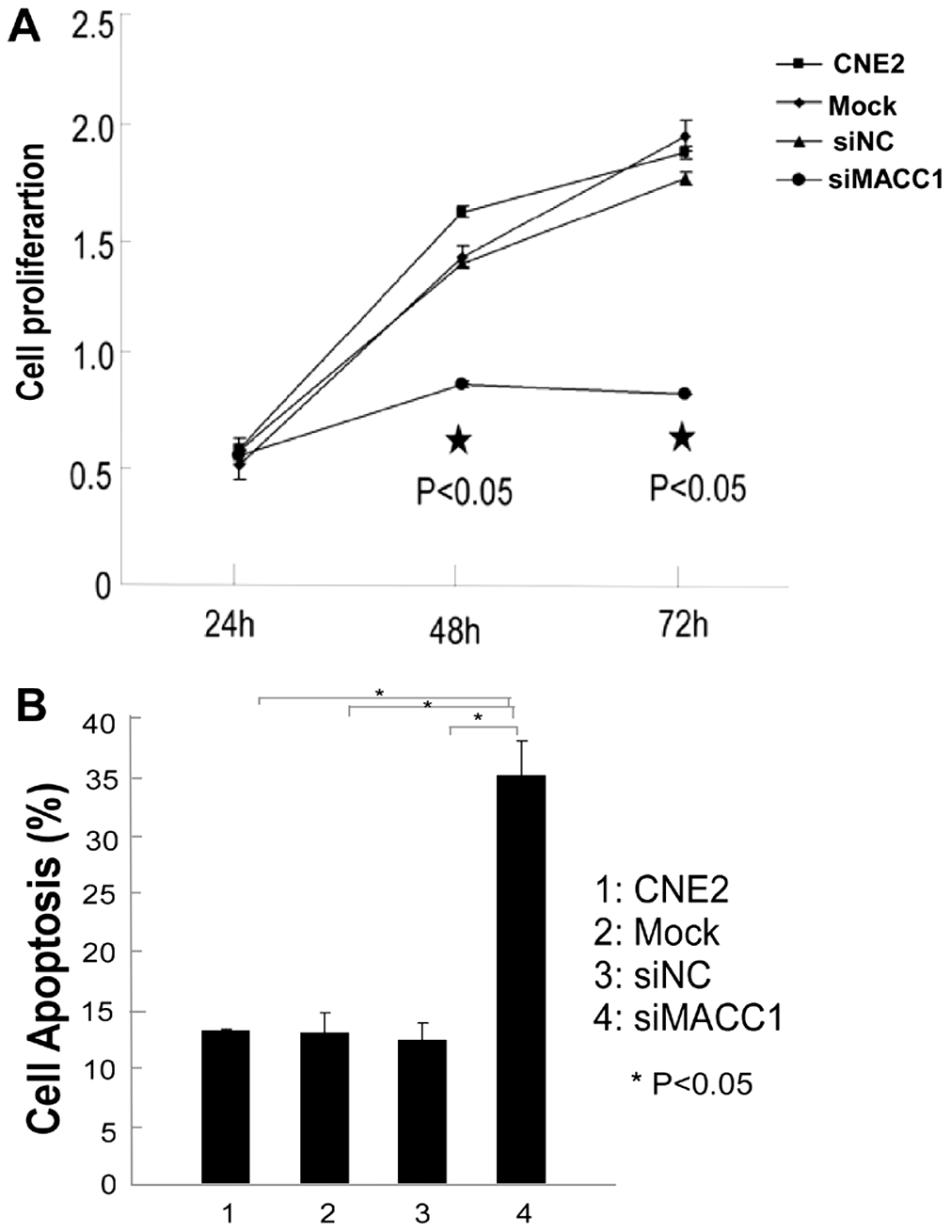
MACC1 knockdown (siMACC1) dramatically suppressed cell proliferation of CNE2 at 48 hours and 72 hours compared with the scramble siRNA(siNC), mock, and untreated CNE2 group by MTT analysis, respectively. *p<0.05 (A), but induced apoptosis of CNE2 transfected with MACC1 siRNA at 48 hours compared with the control group by flow cytometry analysis. *p<0.05(B).

**Figure 3:**
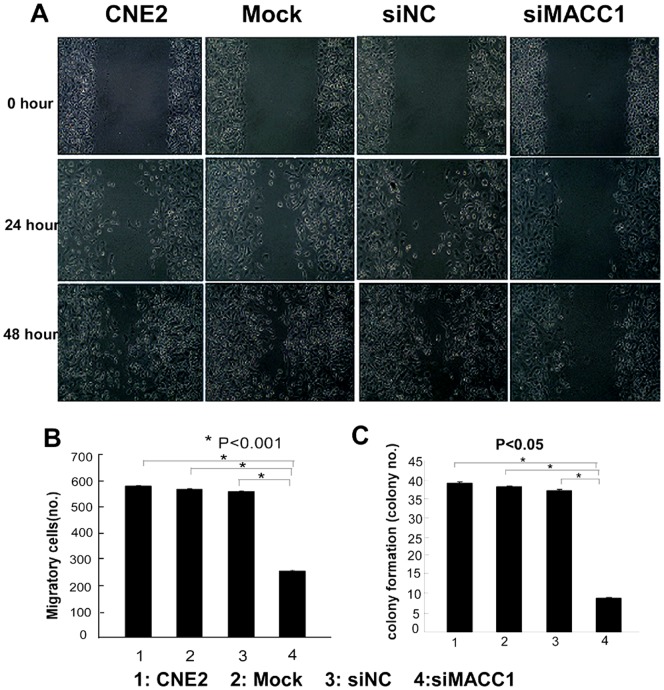
MACC1 knockdown inhibited migration (A), invasion (B) and colony formation (C) of CNE2 compared with control group by scratch wound assay, transwell matrix penetration assay, and colony formation assay, respectively.

**Figure 4:**
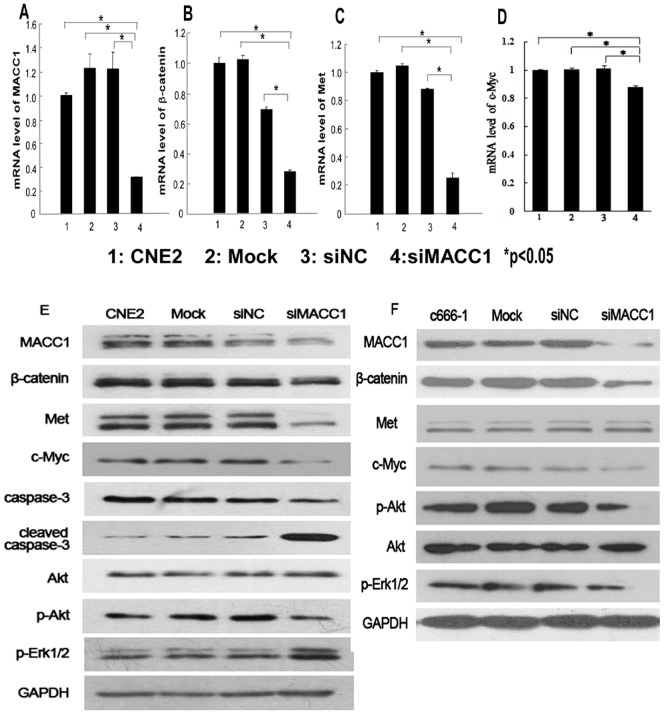
MACC1 down-regulation significantly reduced MACC1, β-catenin, Met, and c-Myc mRNA expression in CNE2 transfected with MACC1 siRNA for 48h by real-time PCR analysis, compared with the control group(A, B, C, D), *p<0.05. At the protein level, MACC1 knockdown resulted in inhibition of β-catenin, Met, and p-Akt (Ser473) protein expression, increased cleaved caspase-3, but no alteration on p-Erk1/2 in CNE2 cell line by western blot analysis (E); MACC1 knockdown resulted in inhibition of β-catenin and p-Akt (Ser473) protein expression, but no alteration on p-Erk1/2 in C666-1 cell line by western blot analysis (F).

To further determine the effect of MACC1 expression on cellular proliferation, apoptosis, migration, invasion and colony formation in NPC cells. CNE2 were transfected with MACC1 siRNA for 48 hours, and 72 hours, the cell proliferation was significantly inhibited in a time-dependent manner compared with the control group by MTT assay (p<0.05, Figure 2). Flow cytometry was used to analyze cell apoptosis in CNE2 transfected with MACC1 siRNA for 48 h. As shown in Figure 2, MACC1 knockdown significantly induced apoptosis in NPC cell line compared with the control group (p<0.05). To further determine whether MACC1 knockdown inhibited migration, invasion and colony formation in NPC cells, the cell migration was significantly suppressed in CNE2 transfected with MACC1 siRNA at 24 hour and 48 hour compared with the control group as shown in Figure 3A. Transwell matrix penetration assay showed that the mean number of invasive CNE2 cell line transfected with MACC1 siRNA group, the scramble siRNA, mock and untreated group at 48 hour was 255, 560, 570, and 582 per field of view, respectively. The difference of cell invasive number between CNE2 transfected with MACC1 siRNA group and the control group was significant (p<0.001, Figure 3B). The cell colony formation assay showed that the mean number of colony formation in CNE2 cell line transfected with MACC1 siRNA group, the scramble siRNA, mock and untreated group was 9, 38, 39, and 40, respectively. The cell colony formation number was significantly suppressed in CNE2 transfected with MACC1 siRNA group at 48 hour compared with the control group (p<0.001, Figure 3C). Meanwhile, western blot analysis showed that full-length caspase-3 which is an important apoptosis effector reduced in CNE2 transfected with MACC1 siRNA compared with the control group, but cleaved caspase-3 elevated in CEN2 transfected with MACC1 siRNA compared with the control group (Figure 4).

### Down-regulation of MACC1 inhibited β-catenin and phosphorylated-Akt expression in NPC cells

To further investigate the underlying mechanism of MACC1 in tumorigenesis of NPC, we down-regulated MACC1 expression in CNE2 cells transfected with MACC1 siRNA for 48 hours , both mRNA and protein levels of β-catenin and Met was significantly suppressed compared with the control group by real-time PCR and western blot analysis, respectively (Figure 4). In addition, mRNA expression of c-Myc was significantly suppressed in treatment group compared with the control group by real-time PCR. However, c-Myc protein level was slightly suppressed in CNE2 cells transfected with MACC1 siRNA. Moreover, we found p-Akt (Ser 473) expression was inhibited in CNE2 transfected with MACC1 siRNA compared with the control group. However, phosphorylated-Erk1/2 (pErk1/2) expression was not altered in CNE2 with MACC1 knockdown (Figure 4). Likewise, we detected these proteins' expression in EBV-positive NPC cell line (C666-1). Our results showed that β-catenin and p-Akt (Ser 473) expression reduced in MACC1 siRNA transfected group, compared with the control groups. However, Met and p-Erk1/2 expression was not altered in C666-1 with MACC1 knockdown (Figure 4).

**Figure 5:**
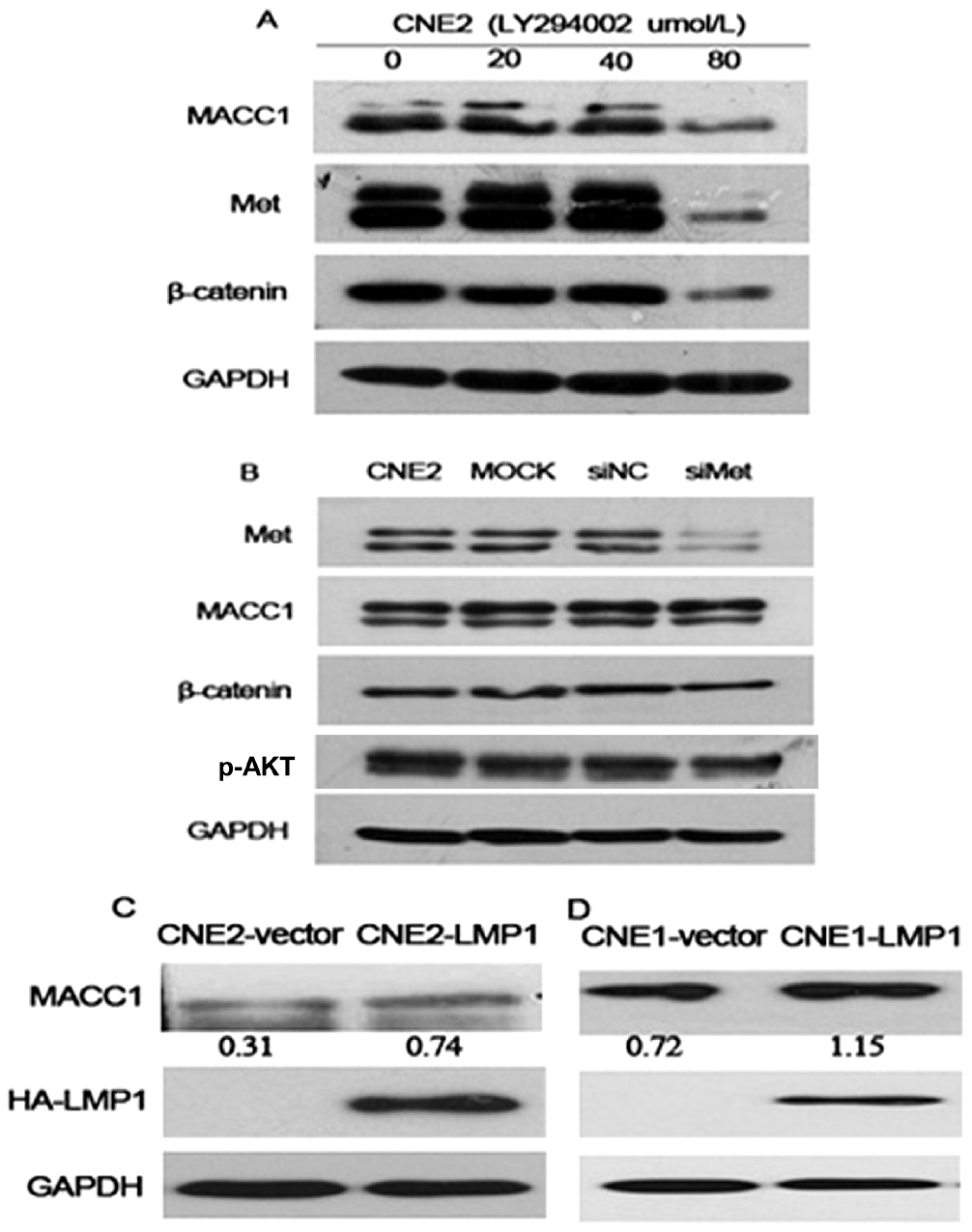
β-catenin and Met expression were inhibited in NPC cells with different concentration of phosphotidylinsitol-3-kinase inhibitor (LY294002) treatment (A); MACC1, β-catenin, and p-Akt expression were not altered in NPC cells transfected with Met siRNA, compared with the control groups (B); LMP1 upregulated MACC1 expression in NPC cell lines- CNE2(C) and CNE1 (D) by western blot analysis. The relative quantification of bands in Western blot was a ratio neutralized to GAPDH.

To further determine the effect of Akt in NPC cells, CNE2 cells were treated with phosphotidylinsitol-3-kinase inhibitor (LY294002, Cell Signaling) at different concentrations (0, 20, 50, 100 µmol/L). Our data demonstrated that β-catenin and Met expression were down-regulated in NPC treatment group. The results suggest that p-Akt regulates β-catenin and Met in NPC cells. Moreover, MACC1, β-catenin and p-Akt expression were not altered in CNE2 transfected with Met-siRNA group, compared with the control groups (Figure 5).

### LMP1 upregulated MACC1 expression in NPC cells

Our above data showed that MACC1 mRNA and protein expression was higher in EBV-positive NPC cell line C666-1 than that in other EBV-negative NPC cell lines, respectively (Figure 1). To further confirm this observation, we constructed LMP1 expression plasmid and transfected LMP1 into EBV-negative cell lines CNE2 and CNE1[5]. Western blot analysis demonstrated that MACC1 expression increased in LMP1-transfected NPC cell line CNE2 and CNE1, compared with the control cells (Figure 5). The results suggest that LMP1 upregulates MACC1 expression in NPC cells. However, the mechanism underlying LMP1 upregulating MACC1 expression in NPC needs further study.

## Discussion

Previous reports have showed that overexpression of MACC1 potentiates metastasis and recurrence of colorectal cancer[7], and associates with peritoneal dissemination and higher stage of TNM classification in colorectal carcinomas[18]. Shirahata et al. have reported that MACC1 expression shows significant correlation with peritoneal dissemination of gastric carcinoma[13]. MACC1 expression may be a useful marker for predicting postoperative recurrence in patients with lung adenocarcinoma after surgery[9], [10]. MACC1 is more frequently expressed in vascular invasive hepatocellular carcinoma[11] and a valuable indicator for stratifying the prognosis of TNM stage I patients with hepatocellular carcinoma[19]. In this study, we firstly showed that MACC1 expression was up-regulated in NPC cell lines compared with the normal nasopharyngeal epithelial cell. Furthermore, immunohistochemistry staining showed that MACC1 expression was higher in NPC tissues than that in chronic inflammation of the nasopharynx. As for the different MACC1 mRNA and protein expression level in HNE1 and SUNE1, the reason may be related to the different regulate factors involving the transcription and translation. Our data showed that elevated expression of MACC1 protein was correlated with the clinical stage and the N classification of NPC.

In order to investigate the role of MACC1 in carcinogenesis of NPC, we down-regulated MACC1 expression in NPC cell line by RNA interference. Our results showed that MACC1 knockdown dramatically inhibited proliferation, migration, invasion and colony formation, but induced apoptosis in NPC CNE2 cells. Zhang et al. have reported that down-regulation of MACC1 by MACC1 specific small hairpin RNA results in significant inhibition of cell proliferation, migration and invasion, meanwhile obvious enhancement of apoptosis in ovarian carcinoma OVCAR-3 cells[12]. These findings suggest that MACC1, as a novel gene, is involved not only in invasion and metastasis, but also in proliferation and apoptosis of carcinomas[8], [20].

Our previous study shows that abnormal expression of β-catenin was found in NPC and β-catenin knockdown suppresses proliferation, migration and invasion of NPC cells[5], [6]. Is there a relationship between MACC1 and β-catenin expression in NPC? Our study showed that there was a significantly positive correlation between MACC1 high expression and β-catenin abnormal expression in NPC (P = 0.003).

MACC1 is a newly identified key regulator of HGF-MET signaling in colorectal carcinoma[8]. Qian et al. have reported Met protein is expressed in NPC and normal nasopharyngeal epithelia. Compared with NPC, the Met expression level is higher in normal columnar nasopharyngeal epithelium but lower in squamous nasopharyngeal epithelium. High Met protein expression level correlates with poorer survival in late-stage NPC[21]. Targeting MET by tyrosine kinase inhibitor suppresses growth and invasion of nasopharyngeal carcinoma cell lines[22]. Silencing of Met by RNA interference inhibits the survival, proliferation, and invasion of nasopharyngeal carcinoma cells[23]. Our present data showed that Met high expression was found in NPC (74.1%) and chronic inflammation of the nasopharynx (91.7%). There was no significant difference between Met expression in NPC and chronic inflammation of the nasopharynx (p>0.05). The results were similar to the previous reports[21]. However, we found that there was a positive correlation between MACC1 expression and Met expression in NPC.

To further clarify the mechanism of MACC1 in carcinogenesis of NPC, we down-regulated MACC1 expression in EBV-negative NPC cell line CNE2 by MACC1 siRNA transfection and found that the expression of Met, p-Akt (Ser473), β-catenin and its downstream gene c-Myc were inhibited, but p-Erk1/2 expression was not affected. Furthermore, We also found p-Akt (Ser473) and β-catenin expression were inhibited in EBV-positive NPC cell line-C666-1 transfected with MACC1 siRNA , but p-Erk1/2 and Met expression was not affected. As for the different Met expression in NPC cell lines with MACC1 siRNA treatment, the reason may be related to the different NPC cell lines, such as different EBV infection status. Stein et al. have reported MACC1 is a key regulator of Met, then further activates MAPK/MEK/Erk signaling pathway, but not PI3K/Akt signaling pathway in colorectal carcinoma[8]. Likewise, in OVCAR-3 cells, down-regulation of MACC1 resulted in expressions of Met, p-MEK1/2, p-ERK1/2, cyclinD1 and MMP2 protein decreased[12]. However, in addition to β-catenin and Met, our results firstly suggest that p-Akt not Erk is the key target gene of MACC1 in NPC. In our present study, there was a significant relationship between MACC1 expression and p-Akt expression (p = 0.03), β-catenin abnormal expression and p-Akt expression (p = 0.012) in NPC tissue, respectively. Further study showed that phosphotidylinsitol-3-kinase inhibitor (LY294002) down-regulated β-catenin and Met expression in NPC cells. Met has also been identified as a transcriptional target of β-catenin[24]. Met and β-catenin pathways are mutually activated in CRC cells[25]. However, MACC1, β-catenin and p-Akt expression were not altered in CNE2 cell transfected with Met-siRNA treatment group, compared with the control groups. In addition, our results showed that LMP1 upregulated MACC1 expression in NPC cells. Our previous data showed that EBV-encoded LMP1 increases nuclear β-catenin accumulation and its transcriptional activity in NPC[5]. β-Catenin knockdown dramatically inhibited cellular growth, migration and invasion, but induced apoptosis of NPC cells[6]. Based on our present data, LMP1/MACC1/Akt/β-catenin pathway maybe plays an important role in carcinogenesis of NPC. The underlined mechanism of this pathway needs further study.

## References

[1] Parkin DM, Whelan S, Ferlay J, Thomas D (2002) Cancer incidence in five continents, vol. VIII. IARC scientific publications. No. 155. Lyon: IARC.

[2] Hildesheim A, Wang CP (2012) Genetic predisposition factors and nasopharyngeal carcinoma risk: A review of epidemiological association studies, 2000–2011: Rosetta Stone for NPC: Genetics, viral infection, and other environmental factors. Semin Cancer Biol 22: 107–116. 22300735 10.1016/j.semcancer.2012.01.007 PMC3296903

[3] Dawson CW, Port RJ, Young LS (2012) The role of the EBV-encoded latent membrane proteins LMP1 and LMP2 in the pathogenesis of nasopharyngeal carcinoma (NPC). Semin Cancer Biol 22: 144–153. 22249143 10.1016/j.semcancer.2012.01.004

[4] White BD, Chien AJ, Dawson DW (2012) Dysregulation of Wnt/beta-Catenin Signaling in Gastrointestinal Cancers. Gastroenterology 142: 219–232. 22155636 10.1053/j.gastro.2011.12.001 PMC3285553

[5] You S, Zhang F, Meng F, Li H, Liu Q, et al.. (2011) EBV-encoded LMP1 increases nuclear beta-catenin accumulation and its transcriptional activity in nasopharyngeal carcinoma. Tumour Biol. 10.1007/s13277-011-0161-x 21336584

[6] Song Y, Yang QX, Zhang F, Meng F, Li H, et al (2012) Suppression of nasopharyngeal carcinoma cell by targeting beta-catenin signalling pathway. Cancer Epidemiol 36: e116–e121. 22142772 10.1016/j.canep.2011.11.002

[7] Stein U, Burock S, Herrmann P, Wendler I, Niederstrasser M, et al (2012) Circulating MACC1 transcripts in colorectal cancer patient plasma predict metastasis and prognosis. PLoS One 7: e49249. 23166620 10.1371/journal.pone.0049249 PMC3498161

[8] Stein U, Walther W, Arlt F, Schwabe H, Smith J, et al (2009) MACC1, a newly identified key regulator of HGF-MET signaling, predicts colon cancer metastasis. Nat Med 15: 59–67. 19098908 10.1038/nm.1889

[9] Shimokawa H, Uramoto H, Onitsuka T, Chundong G, Hanagiri T, et al (2011) Overexpression of MACC1 mRNA in lung adenocarcinoma is associated with postoperative recurrence. J Thorac Cardiovasc Surg 141: 895–898. 21093878 10.1016/j.jtcvs.2010.09.044

[10] Chundong G, Uramoto H, Onitsuka T, Shimokawa H, Iwanami T, et al (2011) Molecular diagnosis of MACC1 status in lung adenocarcinoma by immunohistochemical analysis. Anticancer Res 31: 1141–1145. 21508357

[11] Shirahata A, Fan W, Sakuraba K, Yokomizo K, Goto T, et al (2011) MACC 1 as a marker for vascular invasive hepatocellular carcinoma. Anticancer Res 31: 777–780. 21498695

[12] Zhang R, Shi H, Chen Z, Wu Q, Ren F, et al (2011) Effects of metastasis-associated in colon cancer 1 inhibition by small hairpin RNA on ovarian carcinoma OVCAR-3 cells. J Exp Clin Cancer Res 30: 83. 21923915 10.1186/1756-9966-30-83 PMC3182136

[13] Shirahata A, Sakata M, Kitamura Y, Sakuraba K, Yokomizo K, et al (2010) MACC 1 as a marker for peritoneal-disseminated gastric carcinoma. Anticancer Res 30: 3441–3444. 20944120

[14] Barnes L, Eveson JW, Reichart P, David S (2005) WHO histological classification of tumours of the nasopharynx. World Health Organization classification of Tumours: Pathology and Genetics of Head and Neck Tumours. Lyon: IARC Press. pp. 81–97.

[15] Edge SB, Byrd DR, Carducci MA, Compton CC, Fritz AG, et al.. (2010) TNM classification of carcinomas of the nasopharynx. AJCC cancer staging handbook from the AJCC cancer staging manual. 7th ed ed. NewYork: Springer. pp. 63–79.

[16] Zhang F, Meng F, Li H, Dong Y, Yang W, et al (2011) Suppression of retinoid X receptor alpha and aberrant beta-catenin expression significantly associates with progression of colorectal carcinoma. Eur J Cancer 47: 2060–2067. 21561764 10.1016/j.ejca.2011.04.010

[17] Maruyama K, Ochiai A, Akimoto S, Nakamura S, Baba S, et al (2000) Cytoplasmic beta-catenin accumulation as a predictor of hematogenous metastasis in human colorectal cancer. Oncology 59: 302–309. 11096342 10.1159/000012187

[18] Shirahata A, Shinmura K, Kitamura Y, Sakuraba K, Yokomizo K, et al (2010) MACC1 as a marker for advanced colorectal carcinoma. Anticancer Res 30: 2689–2692. 20682999

[19] Qiu J, Huang P, Liu Q, Hong J, Li B, et al (2011) Identification of MACC1 as a novel prognostic marker in hepatocellular carcinoma. J Transl Med 9: 166. 21955323 10.1186/1479-5876-9-166 PMC3192685

[20] Kokoszynska K, Krynski J, Rychlewski L, Wyrwicz LS (2009) Unexpected domain composition of MACC1 links MET signaling and apoptosis. Acta Biochim Pol 56: 317–323. 19499089

[21] Qian CN, Guo X, Cao B, Kort EJ, Lee CC, et al (2002) Met protein expression level correlates with survival in patients with late-stage nasopharyngeal carcinoma. Cancer Res 62: 589–596. 11809714

[22] Lau PC, Wong EY (2012) Targeting MET by Tyrosine Kinase Inhibitor Suppresses Growth and Invasion of Nasopharyngeal Carcinoma Cell Lines. Pathol Oncol Res 18: 357–363. 21866424 10.1007/s12253-011-9452-1

[23] Li Y, Zhang S, Tang Z, Chen J, Kong W (2011) Silencing of c-Met by RNA interference inhibits the survival, proliferation, and invasion of nasopharyngeal carcinoma cells. Tumour Biol 32: 1217–1224. 21922276 10.1007/s13277-011-0225-y

[24] Boon EM, van der Neut R, van de Wetering M, Clevers H, Pals ST (2002) Wnt signaling regulates expression of the receptor tyrosine kinase met in colorectal cancer. Cancer Res 62: 5126–5128. 12234972

[25] Rasola A, Fassetta M, De Bacco F, D'Alessandro L, Gramaglia D, et al (2007) A positive feedback loop between hepatocyte growth factor receptor and beta-catenin sustains colorectal cancer cell invasive growth. Oncogene 26: 1078–1087. 16953230 10.1038/sj.onc.1209859

